# NT-proBNP predicts the need for ventilatory support in the patients with acute exacerbation of chronic obstructive pulmonary disease

**DOI:** 10.1186/2197-425X-3-S1-A390

**Published:** 2015-10-01

**Authors:** J Kuharic, A Sustic, R Marcun, M Lainscak

**Affiliations:** Dept. of Anesthesiology and ICU, Univ. Hospital Rijeka, Rijeka, Croatia; Dept. of Pneumology, University Clinic of Respiratory Diseases, Golnik, Slovenia; Dept. of Cardiology, General Hospital Celje, Celje, Slovenia

## Introduction

Patients with acute exacerbation of chronic obstructive pulmonary disease (AECOPD) may need ventilatory support (VS) due to respiratory failure. Risk stratification on admission could identify patients at higher risk of deterioration. Cardiac biomarkers are associated with outcome in AECOPD but were not studied as predictors for VS.

## Objectives

The aim of this study was to evaluate association between admission NT-proBNP and ventilatory support (VS) in the patients with AECOPD.

## Methods

The prospective observational study included 139 patients with a clinical diagnosis of AECOPD and Global Initiative for Chronic Obstructive Lung Disease (GOLD) stages III-IV. NT-proBNP was determined from venous blood samples on patient admissions to the hospital with the use of a quantitative electrochemiluminescence assay on an Elecsys 2010 analyzer (Roche Diagnostics) according to established methods. The VS was defined as any form of invasive or noninvasive VS applied during index hospital stay.

## Results

Patients who did not require (no.:108) vs. those who required VS (no.:31) and patients with invasive (no.:15) vs. those with noninvasive VS (no.:16) were of similar age, gender and GOLD stage (p > 0.2 for all). NT-proBNP was higher in patients who required VS then in those without VS (2407 ± 3431 vs. 1709 ± 4648 ng/L; p < 0.05). Patients with noninvasive VS had higher NT-proBNP then those without VS (3213 ± 4389 vs.1709 ± 4648 ng/L; p < 0.05). The difference between patients treated with noninvasive vs. invasive VS was not significant (3213 ± 4389 vs. 1534 ± 1753 ng/L; p=NS). Patients receiving invasive VS had similar admission NT-proBNP as those without VS (1534 ± 1753 vs. 1709 ± 4648 ng/L; p=NS).

## Conclusions

Admission NT-proBNP may predict need for noninvasive VS in patients with AECOPD.

**Figure 1:**
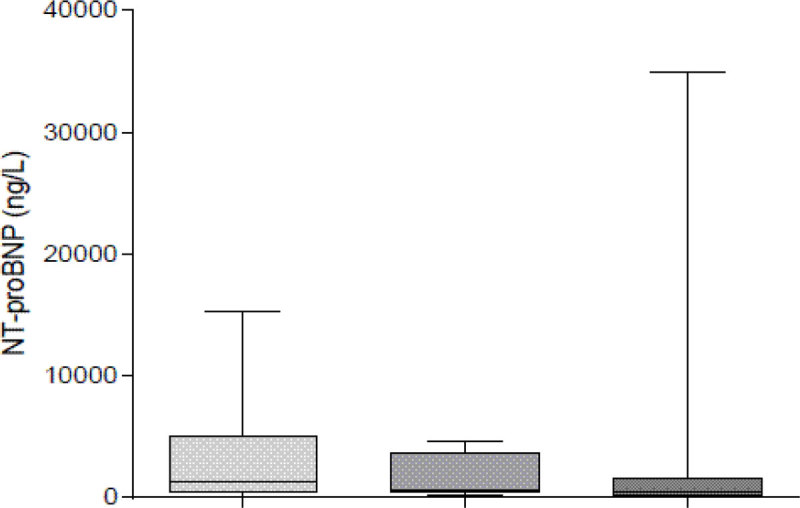
**Relation between NT-proBNP and ventilation.**
